# Multiorgan Dysfunction Caused by Travel-associated African Trypanosomiasis

**DOI:** 10.3201/eid1802.111479

**Published:** 2012-02

**Authors:** Lucy E. Cottle, Joanna R. Peters, Alison Hall, J. Wendi Bailey, Harry A. Noyes, Jane E. Rimington, Nicholas J. Beeching, S. Bertel Squire, Mike B.J. Beadsworth

**Affiliations:** Royal Liverpool University Hospital, Liverpool, UK (L.E. Cottle, J.R. Peters, A. Hall, N.J. Beeching, S.B. Squire, M.B.J. Beadsworth);; Liverpool School of Tropical Medicine, Liverpool (J.W. Bailey, N.J. Beeching, S.B. Squire, M.B.J. Beadsworth);; University of Liverpool, Liverpool (H.A. Noyes);; Hawkshead Medical Practice, Ambleside, UK (J.E. Rimington)

**Keywords:** Trypanosomiasis, African, African sleeping sickness, trypanosomes, parasites, trypanocidal agents, suramin, adverse drug reaction, drug toxicity, tsetse fly, *Trypanosoma brucei rhodesiense*, multi-organ dysfunction, travel, Zambia

## Abstract

We describe a case of multiorgan dysfunction secondary to *Trypanosoma brucei* *rhodesiense* infection acquired on safari in Zambia. This case was one of several recently reported to ProMED-mail in persons who had traveled to this region. Trypanosomiasis remains rare in travelers but should be considered in febrile patients who have returned from trypanosomiasis-endemic areas of Africa.

We describe a British safari tourist with multi-organ dysfunction and shock secondary to African trypanosomiasis. This case illustrates the complications associated with treatment of *Trypanosoma brucei rhodesiense* infection and highlights a recent increase in cases reported to ProMED (www.promedmail.org) of trypanosomiasis in travelers to Zambia.

## The Case-Patient

A 49-year-old woman with a 5-day history of fever, malaise, headache, dizziness, abdominal discomfort, diarrhea, and vomiting sought treatment 1 day after returning to the United Kingdom from a 2-week safari in Zambia. During the safari, she spent 3 days in the South Luangwa National Park, 3 days in the Lower Zambezi National Park, and 6 days in Kafue National Park. Initial blood films examined at Furness General Hospital were negative for malaria parasites but positive for trypanosomes. Urgent transfer of the patient to the Tropical and Infectious Disease Unit in Liverpool, UK, was arranged.

Upon arrival, the patient was dehydrated and had jaundice and tachycardia, but she initially was normotensive. Examination revealed reduced breath sounds at the lung bases and a distended, nontender abdomen. There was a mild erythematous rash on the patient’s abdomen, but no chancres. Results of a neurologic examination were unremarkable.

Repeat blood films confirmed numerous trypanosomes (Figure), which, given the patient’s travel history, were considered likely to be *T. b. rhodesiense*. PCR results confirmed the trypanosomes positive for the *T. b. rhodesiense*–specific serum resistance–associated gene (*1*). Blood test results were as follows: urea 9.2 mmol/L, creatinine 146 μmol/L, leukocytes 3.7 × 10^9^ cells/L, platelets 13 × 10^9^/L, C-reactive protein 234 mg/L, alanine aminotransferase 179 U/L, bilirubin 38 μmol/L, and prothrombin time 13.5 s.

**Figure:**
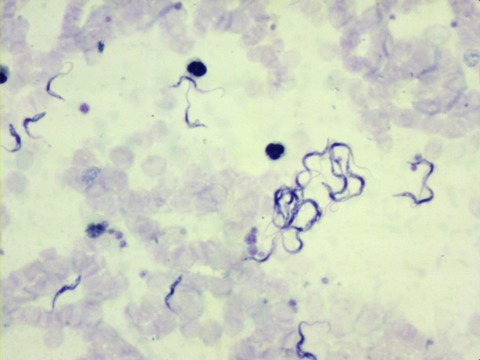
Thick film using Field’s stain showing trypanosomes under ×400 magnification. Motile trypanosomes are shown in the Video.

**Video:**
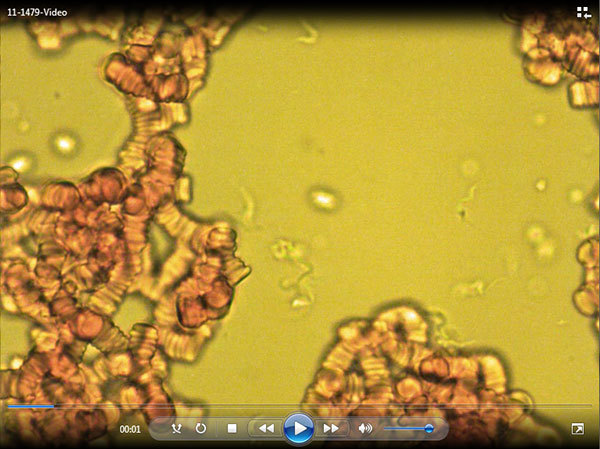
Motile trypanosomes.

Direct Video Link: http://streaming.cdc.gov/vod.php?id=7e517b3f1a94f72aad563c8c5d8ceec720120120160142637

Despite fluid resuscitation, the patient became increasingly hypotensive over the next 12 hours, prompting transfer to the intensive care unit. A 100-mg test dose of suramin was well tolerated by the patient; however, the first full treatment dose was complicated by circulatory collapse and bronchoconstriction, which required administration of hydrocortisone and chlorphenamine and immediate discontinuation of the suramin infusion. Subsequent investigation showed that the suramin dose had been infused more rapidly than prescribed. Further doses administered as slow infusions were uncomplicated.

Hypotension persisted for 4 days but did not necessitate vasopressors. Results of a short synacthen test, electrocardiogram, and echocardiogram were normal.

After the patient received 2 treatment doses of suramin and analysis of repeat blood films confirmed that parasitemia had cleared, a lumbar puncture was performed. The cerebrospinal fluid (CSF) had 4 leukocytes/μL and normal levels of protein and glucose, and no trypanosomes were detected after double centrifugation.

The patient received a full course of suramin for early-stage disease, (regimen in Table 1), which resulted in full recovery. As follow-up care, the patient will receive repeat lumbar punctures every 3 months for 2 years to exclude occult invasion of the central nervous system (CNS). Thus far, 3 repeat lumbar punctures have shown no evidence of CNS invasion.

**Table 1:** Treatment regimen for *Trypanosoma brucei rhodesiense* infection in adults*

Disease stage	Drug, route of administration	Regimen	Adverse effects
First	Suramin, intravenous	Test dose of 100 mg in 100 mL 0.9% saline over 30 min on day 0; and 5 doses of 20 mg/kg (maximum 1 g/dose) in 250 mL 0.9% saline over 3 h on days 1, 3, 7, 14, 21	Hypersensitivity reactions (early and late); nephrotoxicity, hepatotoxicity, hemolytic anemia, peripheral neuropathy, agranulocytosis, thrombocytopenia, and cutaneous reactions
Second	Melarsoprol, intravenous	2.0–3.6 mg/kg/d (maximum 180 mg/d) for 3 d; after 7 d, 3.6 mg/kg/d for 3 d; after 7 more d, 3.6 mg/kg/d for 3 d†	Encephalopathy, cutaneous reactions, peripheral neuropathy, cardiac arrhythmias, thrombophlebitis, fever, and gastric upset

*Source of drug regimen: Brun et al. (*2*) and Abramowicz (*3*). †In frail patients, begin with 18 mg melarsoprol and progressively increase dose (*3*). Pretreatment with suramin for 2–4 d is recommended for debilitated patients.

African trypanosomiasis is caused by the protozoan parasite *T. brucei*, which is transmitted by tsetse flies. Two subspecies are pathogenic in humans: *T. b. gambiense* in central and western Africa, and *T. b. rhodesiense* in eastern and southern Africa.

Disease progresses in 2 stages. In the first stage, parasites spread in the blood to the lymph nodes, liver, spleen, heart, endocrine system, and eyes (*4*). Untreated, they invade the CNS, which leads to second-stage or meningoencephalitic disease with characteristic sleep disturbances. Progression to the second stage may take months in *T. b. gambiense* infection but only weeks in *T. b. rhodesiense* infection.

Although trypanosomiasis is uncommon in travelers, it should be considered in the differential diagnosis of patients with fever who have returned from trypanosomiasis-endemic areas of Africa (*5*). Recent reports suggest an increase in cases emerging from Zambia, particularly from the South Luangwa Valley (Table 2) (*6*). Whether these cases reflect an increased risk for infection in that region or increasing tourism in a trypanosomiasis-endemic area is unclear. Infection in travelers is usually characterized by an acute febrile illness, sometimes associated with a macular evanescent rash or chancre (*2**,**4*). Laboratory tests often indicate anemia, thrombocytopenia, leukopenia, impaired renal function, electrolyte disturbances, coagulation abnormalities, and elevation in hepatic transaminase and C-reactive protein levels (*2**,**7*).

**Table 2:** Reports to ProMED-mail of *Trypanosoma brucei rhodesiense* infections associated with travel to or bordering Zambia, 2010*

Month of report	ProMED-mail archive no.	Nationality of patient	Travel activity	Area visited
September	20100915.3338	Zambian	Visiting game ranch	South Luangwa Valley, Zambia
	20100915.3338	American	Hunting safari	South Luangwa Valley, Zambia
October†	20101022.3833	British	Camping safari	South Luangwa National Park, Lower Zambez National Park, Kafu National Park, Zambia
	20101022.3833	British	Visiting national park	Mana Pool National Park, Zimbabwe (bordering Zambia)
November	20101111.4093	South African national of Scandinavian origin	Hiking	Luangwa River area, Zambia

*Reports in (*6*). Since the start of 2005, 11 other cases of *T. b. rhodesiense* infection in travelers have been reported in ProMED-mail; those cases were acquired in Uganda (1), Tanzania (3), and Malawi (7). †Case presented in this report.

Conditions that should be considered in patients with persistent hypotension are adrenal insufficiency and cardiac dysfunction. The prevalence of adrenal insufficiency was 27% in a study of Ugandan patients with trypanosomiasis (*8*). Myocarditis, pericarditis, and congestive cardiac failure have been described and should be excluded by electrocardiogram and echocardiography (*4*).

The treatment for first-stage *T. b. rhodesiense* infection is intravenous suramin, given as 5 injections of 20 mg/kg each over 3–4 weeks (*2**,**4*). Early hypersensitivity reactions to suramin (i.e., nausea, circulatory collapse, and urticaria) are described in 0.1%–0.3% of patients; thus, an initial test dose is advocated (*9*).

Second-stage *T. b. rhodesiense* infection is treated with melarsoprol, a highly toxic arsenical which causes a severe reactive encephalopathy in ≈10% of patients, half of whom die as a result (*10*). This toxicity among patients emphasizes the importance of accurate staging, which is determined by CSF examination. According to World Health Organization guidelines, the presence of >5 leukocytes/μL and/or the presence of trypanosomes in the CSF indicates second-stage disease (*11*). Lumbar puncture should be deferred until clearance of blood parasitemia has been confirmed.

In view of our patient’s rapid onset of a high level of parasitemia, we investigated the possibility of a genetic susceptibility to trypanosomal infection. Human plasma contains a trypanosome lytic factor called apolipoprotein L-1 (APOL1) (*12*). This protein causes lysis of *T. brucei* subspecies other than *rhodesiense* and *gambiense,* both of which have acquired resistance to it (*13*). In 2006, Vanhollebeke et al. (*14*) described a patient infected with *T. evansi*, which is usually sensitive to APOL1. The patient’s serum lacked APOL1 due to mutations in the *APOL1* gene, rendering him susceptible to a species regarded as nonpathogenic in humans. We sequenced the *APOL1* gene of our patient, but no substantial variations suggesting enhanced susceptibility were detected.

## Conclusions

In summary, trypanosomiasis remains rare in travelers, but possible infection should be considered in patients with fever who have returned from trypanosomiasis-endemic areas of Africa. Early reporting of trypanosomiasis cases to ProMED-mail allows timely recognition of emerging safari destinations that present an increased risk for infection to travelers. In patients with *T. b. rhodesiense* infection, multi-organ dysfunction may develop in early-stage disease. Treatment of such cases should be managed with critical care support, and it should be remembered that rapid infusion of suramin may precipitate circulatory collapse.
